# Use of computerized campimetry and/or optical coherence tomography for glaucoma diagnosis by non-glaucoma specialists

**DOI:** 10.5935/0004-2749.20210016

**Published:** 2025-02-02

**Authors:** Cláudia Gomide Vilela de Sousa Franco, Marcos Pereira de Ávila, Leopoldo Magacho

**Affiliations:** 1 Ophthalmology Department, Universidade Federal de Goiás, Goiânia, GO, Brazil; 2 VER-Excellence in Ophthalmology, Goiânia, GO, Brazil

**Keywords:** Glaucoma, Tomography, optical coherence, Visual field tests, Optic disk, Observer variation, Glaucoma, Tomografia de coerência óptica, Testes de campo visual, Disco óptico, Variações dependentes do observador

## Abstract

**Purpose:**

To compare the use of visual field and/ or optical coherence tomography (OCT) combined with color retinography by non-glaucoma specialists for differentiating glaucoma from physiological cupping.

**Methods:**

Eighty patients with glaucoma or physiological cupping (40 of each) were randomized according to the examination used (GI: color retinography, GII: color retinography + visual field, GIII: color retinography + optical coherence tomography, GIV: color retinography + visual field + optical coherence tomography). Twenty non-specialist ophthalmologists diagnosed glaucoma from PowerPoint slide images, without direct patient examination.

**Results:**

Inter-examiner agreement was good for GII (ĸ: 0.63; 95%CI, 0.53-0.72), moderate for GIII (ĸ: 0.58; 95%CI, 0.48-0.68) and GIV (ĸ: 0.41; 95%CI, 0.31-0.51), and low for GI (ĸ: 0.30; 95%CI, 0.20-0.39) (p<0.001). Diagnostic accuracy was higher in GIII (15.8 ± 1.82) than GI (12.95 ± 1.46, p<0.001) and higher in GII (16.25 ± 2.02) than GI and GIV (14.10 ± 2.24) (both p<0.001). For glaucoma patients only, diagnostic accuracy in GII and GIII was superior to that in GI and GIV (both p<0.001). Sensitivity and specificity were 59% and 70.5% in GI; 86.5% and 76% in GII, 86.5% and 71.5% in GIII; and 68.5% and 72.5% in GIV, respectively. Accuracy was highest in GII (81.3% [95%CI, 77.1-84.8]), followed by GIII (79% [95%CI, 74.7-82.7]), GIV (70,5% [95%CI, 65.9-74.8]), and GI (64.8% [95%CI, 60.0-69.3]).

**Conclusions:**

Non-glaucoma specialists could not differentiate glaucoma from increased physiological cupping when using color retinography assessment alone. Diagnostic accuracy and inter-rater agreement improved significantly with the addition of visual field or optical coherence tomography. However, the use of both modalities did not improve sensitivity/specificity.

## Abstract (pt)

**Objetivos:**

Verificar a influência do campo visual e/ou tomografia de coerência óptica, quando analisados em associação à retinografia colorida, na diferenciação entre indivíduos com glaucoma daqueles com aumento fisiológico de escavação.

**Métodos:**

Oitenta pacientes com glaucoma ou aumento fisiológico de escavação (40 cada) foram randomizados de acordo com o exame testado (GI: retinografia colorida, GII: retinografia colorida + campo visual, GIII: retinografia colorida + tomografia de coerência óptica, GIV: retinografia colorida + campo visual + tomografia de coerência óptica). Vinte oftalmologistas não especialistas em glaucoma diagnosticaram glaucoma através de slides do PowerPoint, sem o exame direto do paciente.

**Resultados:**

A concordância interexaminador foi boa para o GII (ĸ: 0,63; 95%CI, 0,53-0,72), moderada para GIII (ĸ: 0,58; 95%CI, 0,48-0,68) e GIV (ĸ: 0,41; 95%CI, 0,31-0,51), e baixa para o GI (ĸ: 0,30; 95%CI, 0,20-0,39) (p<0,001). Acurácia diagnostica foi maior no GIII (15,8 ± 1,82) em comparação ao GI (12,95 ± 1,46, p<0,001) e o GII (16,25 ± 2,02) maior em comparação ao GI e GIV (14,10 ± 2,24) (para ambos, p<0,001). Para os pacientes com glaucoma, a acurácia diagnostica nos grupos GII e GIII foi superior do que em GI e GIV (ambos p<0,001). Sensibilidade e especificidade foram 59% e 70,5% no GI; 86,5% e 76% no GII, 86,5% e 71,5% no GIII; 68,5% e 72,5% no GIV, respectivamente. A acurácia foi maior no GII (81,3% [95%CI, 77,1-84,8]), seguido pelo GIII (79% [95%CI, 74,7-82,7]), GIV (70,5% [95%CI, 65,9-74,8]), e GI (64,8% [95%CI, 60,0-69,3]).

**Conclusões:**

A avaliação isolada da retinografia colorida por oftalmologistas não especialistas em glaucoma não pode diferenciar pacientes com glaucoma daqueles com aumento fisiológico de escavação. Houve aumento da acurácia diagnóstica e da concordância interobservador com o acréscimo do campo visual ou da tomografia de coerência óptica. Entretanto, o uso de ambas as modalidades não melhorou a sensibilidade/especificadade.

## INTRODUCTION

Glaucoma is a chronic optical neuropathy characterized by damage to the optic disc (OD) and the retinal nerve fiber layer (RNFL). This damage usually results in corresponding functional loss in visual field (VF) changes^(1)^. According to the World Health Organization (WHO), glaucoma is the leading cause of irreversible blindness worldwide and the second most common cause after cataracts when reversible causes of blindness are taken into consideration^(2)^.

OD and RNFL assessments show a large inter-individual variability with age, sex, ethnicity, and refractive error^(3)^. Quigley et al. suggested that functional damage to the OD, as assessed through VF changes, would occur after the loss of between 40% and 50% of retinal ganglion cells (RGCs), which is usually related to structural damage^(4)^. On the other hand, data from large clinical trials have shown that damage to the perimetry precedes OD changes during the progression of glaucoma^(5,6)^. However, the detection of both structural and functional changes may occur simultaneously in some patients, while either structural or functional changes may occur first in other patients^(7)^.

The diagnostic ability of complementary tests to evaluate the OD in order to detect glaucomatous loss is comparable to an OD examination by glaucoma specialists, as reported in the first consensus statement of the Association of International Glaucoma Societies^(8)^. However, a considerable proportion of glaucoma patients are cared for by non-glaucoma specialists. There are no data regarding the impact of the complementary examinations used for glaucoma diagnosis by these ophthalmologists. Moreover, it is important to know whether a single test or a combination of tests may lead to an increased ability to diagnose glaucoma. Here, we addressed these gaps by comparing the use, by non-glaucoma specialists, of VF and/or optical coherence tomography (OCT) combined with color retinography (CR) for differentiating between glaucoma and physsiological cupping.

## METHODS

Eighty patients who attended the Hospital VER-Excellence in Ophthalmology, participated in this study. Approval for the study was provided by the Independent Ethics Committee of Hospital VER and the Independent Ethics Committee of the Federal University of Goiás (CAAE 55524316.2.0000.5078).

For all patients, SITA standard 24-2 VF tests (Humphrey Systems, Dublin, CA, USA), digital CR (Visucam Lite, Carl Zeiss Meditec, Jena, Germany), and RTVue OCT (Optovue Inc., Fremont, CA, USA) analyses were performed by the same trained and experienced technician. Only reliable tests of the right eye, performed at no more than 7-day intervals, were taken into consideration. VF was considered only if it had fixation losses of <20%, false positives of <33%, and false negati ves of <33%^(9)^. For OCT, only well-centered images with a signal strength intensity of ≥30 were included^(10)^.

Chart analysis and patient enrollment were performed retrospectively and consecutively from January 2013. Patients were randomized into four groups, with 20 patients per group. Each group contained ten patients with glaucoma and ten patients with suspected glaucoma (defined here as physiological cupping). These latter patients did not have intraocular pressure (IOP) >21 mmHg or other signs of glaucoma, as described below.

Inclusion criteria included the ability to perform a VF test at least in the right eye and a best corrected visual acuity of ≥20/30. Patients’ most recent VF test results were selected. Patients were considered to have glau coma if they had characteristic signs in the OD and based on the analysis of the ganglion cell complex (GCC) using OCT protocols, including the RNFL, ganglion cell layer, and inner plexiform layer^(11)^.

Patients with suspected glaucoma were eligible if they had no history of any eye disease; did not show IOP increase, glaucomatous OD, and/or RNFL suggestive of glaucoma; and had reliable VF and OCT, according to the criteria described above. Moreover, all examinations submitted to the examiners had to correspond to their normal or change group.

Exclusion criteria for both groups were: vision loss or deficit in either eye from an unknown disease other than glaucoma; recent intraocular surgery (within the last 3 months); unreliable VF; any other change to biomicroscopy or color fundus photography that could interfere with VF and/or OCT evaluations, or recent participation in another study protocol (within the last 6 months).

The diagnosis of glaucoma or physiological cupping was made by two glaucoma specialists with access to all data in a patient’s records (L.M., C.G.).

A patient was considered to have glaucoma if their eye(s) had at least one of the following characteristics besides an increased C/D ratio (>0.6): loss of the ISNT, localized thinning at the neural border with vessel changes, RNFL wedge-shaped defect (Hoyt’s signal), or the presence of a peridiscal beta zone. Hodapp-Parrish-Anderson criteria were considered for VF diagnosis^(12)^.

**Figure 1:**
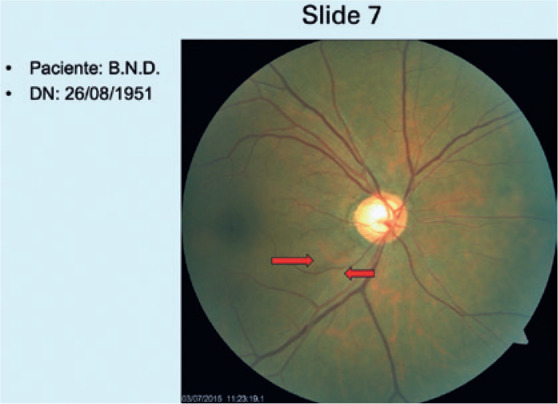
Example of a slide from a patient in GI (slides 1 to 20), in which only the patient’s CR image, initials, and date of birth are displayed. In this example case, the patient had glaucoma. Hoyt signal (red arrows).

**Figure 2:**
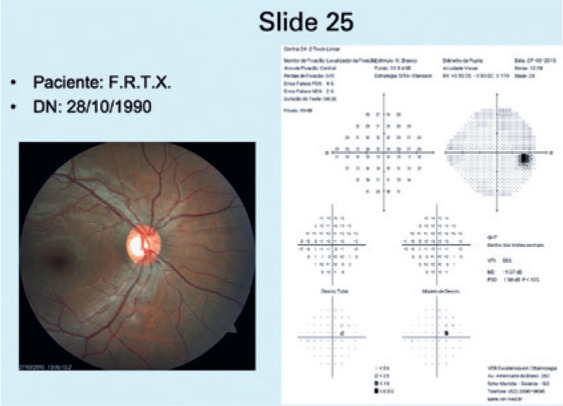
Example of a slide from a patient in GII (slides 21 to 40), in which only the patient’s CR and VF images, initials, and date of birth are displayed. In this example case, the patient had physiological cupping.

**Figure 3:**
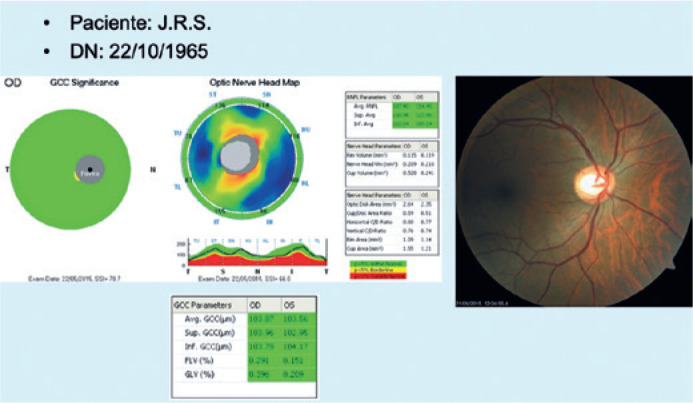
Example of a slide from a patient in GIII (slides 41 to 60), in which only the patient’s CR and OCT images, initials, and date of birth are displayed. In this example case, the patient had physiological cupping.

**Figure 4:**
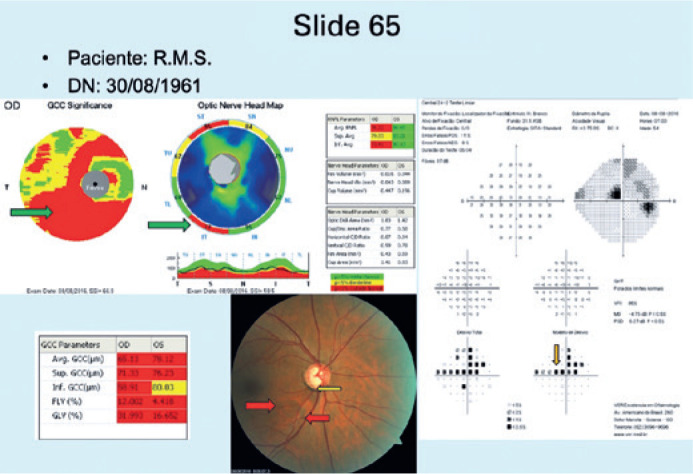
Example of a slide from a patient in GIV (slides 61 to 80), in which only the patient’s CR, VF, and OCT images, initials, and date of birth are displayed. In this example case, the patient had glaucoma. Hoyt signal (red arrows), notching (yellow arrow), upper paracentral scotoma on the Pattern Deviation Probability chart (orange arrow), with corresponding macular ganglion cell loss and thinning of the peripapillary retinal nerve fiber layer (green arrows).

Images of these eyes were prepared in PowerPoint. For the first group (GI), the slides showed only CR images (Figure 1). For the second group (GII), the slides showed images from CR+VF (Figure 2). For the third group (GIII), the slides showed images of CR+OCT evaluations (Figure 3). Lastly, for the fourth group (GIV), CR+VF+OCT images were shown (Figure 4).

CR= color retinography; OD= optic disk.

CR= color retinography; VF= visual field.

CR= color retinography; OCT= optical coherency tomography.

CR= color retinography; VF= visual field; OCT= optical coherency tomography.

All images were sent within a single PowerPoint file (Microsoft Office Professional Plus 2010) via email to 20 non-glaucoma specialists. An appropriate time for assessment was allowed. All examiners signed an informed consent form before evaluating the slides. The order in which slides were shown was always the same; this order was randomly generated using the website *www.randomization.com*. The same website was used to allocate patients to the different groups.

All examiners were informed that half of the patients had glaucoma, while the other half were suspected of having glaucoma. All ophthalmologists were instructed to define whether the patient had glaucoma or not, using an attached Excel table, which contained only the patient numbers.

Statistical analyses were performed using SPSS software, version 22.0 (Statistical Package for the Social Sciences; SPSS Inc, Chicago, IL, USA). In order to check the normality of the distribution, Kolmogorov-Smirnov and Shapiro-Wilk tests were applied. For both tests, variables with p>0.05 were considered as being within normal values and thus having a normal distribution. The Kruskal-Wallis test was used to compare non-parametric quantitative variables among the four study groups. For statistically significant differences, a multiple comparison test was applied. Kappa statistics were used to conduct a concordance analysis between the correct diagnosis and the answer of each physician for each group^(13).^

The estimation of sensitivity (Se), specificity (Sp), positive-predictive value (PPV), negative-predictive value (NPV), and accuracy of each group, with its corresponding *kappa* concordance and 95% confidence intervals, was performed using OpenEpi software (Dean AG, Sullivan KM, Soe MM. *Open Source Epidemiologic Statistics for Public Health*, *Version 3. www.OpenEpi.com, updated on 04/06/2013).* Qualitative variables were shown as per frequency distributions, and the chi-square test and Fisher’s exact test were applied. A significance level of 5% (p<0.05) was used.

## RESULTS

There were 1600 assessments, 400 per group, for 80 eyes of 80 patients, with 20 patients per group, performed by 20 ophthalmologists. The study population comprised 67.5% (54) females, with no statistically significant difference in sex distribution between glaucoma vs. physiological cupping patients: 70% (28) and 65% (26), respectively (p=0.6).

The mean age in the glaucoma group was higher than in the physiological cupping group (65.23 ± 12.66 vs. 48.48 ± 13.77, p<0.001). There was no statistically significant difference in mean age among the four groups (p=0.9).

When analyzing the VF parameters in the patients with glaucoma from GII and GIV, there was a statistically significant difference only for pattern standard deviation (PSD), p=0.04 (Table 1). There were no statistically significant differences in the VF indices between the patients with physiological cupping in GII and GIV. When comparing VF parameters between physiological cupping and glaucoma patients in GII, there were significant differences only for mean deviation (MD) rates (-0.73 ± 0.95 vs. -9.66 ± 5.45, p<0.001) and PSD (1.74 ± 0.54 vs. 9.08 ± 3.25, p<0.001).

**Table 1:** Comparison of mean VF rates among patients with glaucoma in GII (n=10) and GIV (n=10)

	Unit	Group II	Group IV	P
**MD**	dB	-9.66 ± 5.45	-6.67 ± 4.70	0.2
**PSD**	dB	9.08 ± 3.25	5.78 ± 3.33	0.04
**FP**	%	2.70 ± 4.19	3.80 ± 2.97	0.09
**FN**	%	4.20 ± 5.09	8.20 ± 12.11	0.6
**FL**	n°	0.10 ± 0.31	0.30 ± 0.94	0.9
**VFI**	%	71 ±16.07	83.6 ±14.19	0.07

dB= decibel; MD= mean deviation; PSD= pattern standard deviation; FP= false positive; FN= false negative; FL= fixation losses; CR= color retinography; VF= visual field; VFI= visual field index; OCT= optical coherence tomography. GII= CR + VF; GIV= CR + VF + OCT.

There were no statistically significant differences in the OCT parameters when comparing patients with glaucoma between GIII and GIV (Table 2). The descriptive analyses of GIII and GIV are shown in tables 3 and 4, respectively.

**Table 2:** Comparison of mean OCT parameters among patients with glaucoma in GIII (n=10 patients) and GIV (n=10 patients)

	Unit	Group III	Group IV	P
**Avg. RNFL**	µm	77.79 ± 12.75	95.31 ± 34.86	0.1
**Sup. RNFL**	µm	79.50 ± 13.52	92.50 ± 26.48	0.1
**Inf. RNFL**	µm	76.07 ± 12.61	98.12 ± 43.63	0.1
**Vertical C/D**	N/A	0.91 ± 0.09	0.84 ± 0.2	0.2
**Avg. CCG**	µm	80.78 ± 13.99	83.59 ± 19.17	0.7
**Sup. CCG**	µm	79.57 ± 14.13	84.38 ± 14.9	0.4
**Inf. CCG**	µm	82.05 ± 17.34	82.82 ± 24.60	0.7
**FLV**	%	6.03 ± 4.09	7.43 ± 5.03	0.4
**GLV**	%	17.96 ± 11.99	18.4 ± 8.68	0.7

Avg RNFL= mean retinal nerve fiber layer thickness; Sup RNFL= mean upper retinal nerve fiber layer thickness; Inf RNFL= mean lower retinal nerve fiber layer thickness; Vertical C/D= cupping/vertical OD relationship; CCG= ganglion cell complex; Avg CCG= mean CCG thickness; Sup CCG= mean upper CCG thickness; Inf CCG= mean lower CCG thickness; FLV= focal loss volume; GLV= global loss volume; µm= micrometer; N/A= not applicable; CR= color retinography; VF= visual field; VFI= visual field index; OCT= optical coherence tomography. GIII= CR + OCT; GIV= CR + VF + OCT.

**Table 3:** Comparison of mean OCT parameters among patients with physiological cupping vs. patients with glaucoma in GIII

	Unit	Suspects	Glaucoma	P
**Avg. RNFL**	µm	103.06 ± 13.79	77.79 ± 12.75	0.001
**Sup. RNFL**	µm	100.52 ± 14.27	79.50 ± 13.52	0.004
**Inf. RNFL**	µm	105.59 ± 14.72	76.07 ± 12.61	<0.001
**Vertical C/D**	N/A	0.81 ± 0.09	0.91 ± 0.09	0.02
**Avg. CCG**	µm	93.15 ± 9.09	80.78 ± 13.99	0.03
**Sup. CCG**	µm	92.66 ± 9.69	79.57 ± 14.13	0.02
**Inf. CCG**	µm	93.65 ± 8.70	82.05 ± 17.34	0.07
**FLV**	%	0.45 ± 0.41	6.03 ± 4.09	0.005
**GLV**	%	5.74 ± 4.94	17.96 ± 11.99	0.005

Avg RNFL= mean retinal nerve fiber layer thickness; Sup RNFL= mean upper retinal nerve fiber layer thickness; Inf RNFL= mean lower retinal nerve fiber layer thickness; Vertical C/D= cupping/vertical OD relationship; CCG= ganglion cell complex; Avg CCG= mean CCG thickness; Sup CCG= mean upper CCG thickness; Inf CCG= mean lower CCG thickness; FLV= focal loss volume; GLV= global loss volume; µm= micrometer; N/A= not applicable; CR= color retinography; VF= visual field; VFI= visual field index; OCT= optical coherence tomography. GIII= CR + OCT.

**Table 4:** Comparison of mean OCT and VF testing parameters among patients with physiological cupping and those with glaucoma in GIV

	Unit	Suspects	Glaucoma	P
**Avg. RNFL**	µm	99.62 ± 13.68	95.31 ± 34.86	0.1
**Sup. RNFL**	µm	98.87 ± 17.33	92.50 ± 26.48	0.2
**Inf. RNFL**	µm	100.37 ± 10.77	98.12 ± 43.63	0.1
**Vertical C/D**	N/A	0.83 ± 0.09	0.84 ± 0.2	0.3
**Avg. CCG**	µm	87.18 ± 7.49	83.59 ± 19.17	0.5
**Sup. CCG**	µm	86.3 ± 8.01	84.38 ± 14.9	0.7
**Inf. CCG**	µm	88.16 ± 7.59	82.82 ± 24.60	0.1
**FLV**	%	1.12 ± 0.92	7.43 ± 5.03	<0.001
**GLV**	%	9.59 ± 6.27	18.4 ± 8.68	0.04
**MD**	Db	-1.35 ± 1.07	-6.67 ± 4.70	0.002
**PSD**	dB	1.68 ± 0.34	5.78 ± 3.33	<0.001
**FP**	%	3.2 ± 2.53	3.80 ± 2.97	0.7
**FN**	%	1.2 ± 1.81	8.20 ± 12.11	0.1
**PF**	n°	0.10 ± 0.31	0.30 ± 0.94	0.9
**VFI**	%	99.10±0.73	83.6±14.19	<0.001

Avg RNFL= mean retinal nerve fiber layer thickness; Sup RNFL= mean upper retinal nerve fiber layer thickness; Inf RNFL= mean lower retinal nerve fiber layer thickness; Vertical C/D= cupping/vertical OD relationship; CCG= ganglion cell complex; Avg CCG= mean CCG thickness; Sup CCG= mean upper CCG thickness; Inf CCG= mean lower CCG thickness; FLV= focal loss volume; GLV= global loss volume; µm= micrometer; N/A= not applicable; CR= color retinography; VF= visual field; VFI= visual field index; OCT= optical coherence tomography. GIV= CR + VF + OCT.

Correct diagnoses differed significantly between the groups: an accurate diagnosis was higher in GIII (15.8 ± 1.82) than in GI (12.95 ± 1.46) (p <0.001); it was also higher in GII (16.25 ± 2.02) than in GI and GIV (14.10 ± 2.24, p<0.001 for both). There were no significant differences in the numbers of correct diagnoses between GI and GIV (p=0.5), between GIII and GIV (p=0.1), or between GII and GIII (p=1.0). Considering the slides of glaucoma patients only, GII and GIII performed better than GI and GIV (p<0.001). There were no significant differences between GI and GIV (p=0.5) or between GII and GIII (p=1.0). There were no statistically significant differences in the number of correct diagnoses using slides of patients with physiological cupping among the four groups (p=0.5).

Se, Sp, PPV, NPV, and accuracy were higher in GII (86.5%, 76.0%, 78.3%, 84.9%, 81.3%, respectively), followed by GIII (86.5%, 71.5%, 75.2%, 84.1%, 79.0%, respectively), and GIV (68.5%, 72.5%, 71.4%, 69.7%, 70.5% respectively), with the lowest in GI (59.0%, 70.5%, 66.7%, 63.2%, 64.8%, respectively).

There were significant differences (p<0.001) in the intra-observer concordance coefficient (kappa) among the groups, with the highest rate in GII (κ, 0.63; 95% CI, 0.53-0.72), followed by GIII (κ, 0.58; 95% CI, 0.48-0.68), GIV (κ, 0.41; 95% CI, 0.31-0.51), and GI (κ, 0.30; 95% IC, 0.20-0.39).

## DISCUSSION

In the present study, we found that the combination of VF and CR increased the Se (86.5% vs. 59%), Sp (76% vs. 70.5%), accuracy (81.3% vs. 64.8%), PPV (78.3% vs. 66.7%), and NPV (84.9% vs. 63.2%) of glaucoma diagnoses by non-glaucoma specialists compared with these factors when using CR analysis alone. Similar results were obtained when OCT was combined with CR compared with the results when using CR alone. However, when all three tests were used together (CR + VF + OCT), no increase in the rate of correct diagnoses was observed, although the standard deviation increased.

OD assessment alone for glaucoma diagnosis is poorly reproducible, due to decreased concordance, even among specialists^(14)^. This is mainly due to the subjectivity of the examination and the marked variability in OD morphology seen, even among healthy individuals.

In this study, a lower rate of concordance among ophthalmologists and a lower rate of correct diagnoses were found in GI (ĸ: 0.30; 12.95 ± 1.46). This group also showed a lower Se (59%) and Sp (70.5%) for glaucoma diagnosis.

The poor diagnostic ability of CR for glaucoma may relate to the one-off nature of the assessment, rather than being a longitudinal assessment. Additionally, OD dimensions were not assessed to ascertain their impact on the final results in each group. The establishment of a standardized methodology to assess OD and RNFL through CR, in addition to the possibility of using contralateral eye analysis for cupping asymmetry assessment, could increase concordance among observers and consequently the probability of a correct diagnosis. It is possible that the concurrent use of “red-free” retinographies could lead to Se/Sp improvement, since RNFL defects could be better evidenced^(15)^.

The control group was composed of patients with physiological cupping, which may have hampered correct identification, mainly in GI (CR only). In a setting where the control group comprised only ODs with a fully physiological appearance, a small, regular C/D ratio, and the absence of RNFL defects and vascular changes, the Sp may have been artificially increased. However, the presence of ODs showing normal characteristics, despite cupping, is very important, as these discs can typically lead to uncertainty in glaucoma diagnosis. These controls were specifically included to avoid an important type of control group bias (“spectrum bias”)^(16,17)^.

Despite the poor consensus for initial glaucoma diagnosis, most specialists agree that the presence of structural damage is crucial, whereas loss of VF, as assessed by the VF test, may be used to increase the probability of correct disease diagnosis. According to the World Glaucoma Association consensus for Open-Angle Glaucoma diagnosis, a combination of structural assessment plus VF 24-2, with outside normal limits, significantly increases the chance of a glaucoma diagnosis^(18)^. Similar results were obtained in the present study when the VF test was added to the assessment.

In the present study, a larger concordance among examiners was found in GII (ĸ, 0.63), followed by GIII (ĸ, 0.58), GIV (ĸ, 0.41), and GI (ĸ, 0.3). It is possible that the VF test, which is more widely used among ophthalmologists, even non-glaucoma specialists, was the reason for this finding. Even without OCT, inclusion of the VF test improved both the rate of correct diagnoses and concordance among examiners, suggesting that the addition of a second complementary test is important for glaucoma assessment.

The Se of a glaucoma diagnosis using CR only increases considerably as the severity of functional loss increases^(19)^. Thus, it is possible that greater glaucoma severity in patients from GII than from GIV (PSD: 9.08 ± 3.25 vs. 5.78 ± 3.33, p=0.04) could have artificially increased the Se/Sp in the former group. In GIV, OCT analysis was available; this test may show changes in the early stages of disease, while the VF 24-2 results are still normal^(20)^. Thus, the low diagnostic performance seen with OCT increase, as compared with the results of previous studies^(21,22)^, may be explained, at least in part, by the difference in severity among glaucoma patients in these groups, with more patients with early disease stages enrolled in GIV, which influenced the difference in final correct diagnoses. However, even in GIV, patients on average had moderate glaucoma (MD: -6.67 ± 4.70 dB).

A recent study showed that OCT Sp was the parameter most affected by the standard reference test used for glaucoma diagnosis, and this was slightly higher when the diagnostic reference used was CR^(21)^. In the current study, we included general ophthalmologists only and not glaucoma specialists; these clinicians essentially used OD characteristics to determine whether the examined image was healthy, since OCT-based structural assessment did not lead to a significant increase in the GIII Sp.

There was an increase in Se (86.5% vs. 59%), Sp (71.5% vs. 70.5%), and accuracy (79% vs. 64.8%) when OCT was combined with OD/RNFL analysis (GIII) compared with these parameters when using CR evaluation alone (GI) (p<0.001). This result agreed with the findings of previous studies that reported an increase in diagnostic Se by general ophthalmologists when structural analysis based on imaging was combined with a subjective OD evaluation^(23,24)^.

Imaging with RTVue in this study used the ONH and GCC protocols. These protocols map the distribution of the RNFL around the OD and provide a sectorial measure, a map of GCC significance, and global values^(25)^.

Oddone et al. found a low Se of glaucoma diagnosis when OCT alone was used, even based on the best parameter (inferior peripapillary RNFL thickness) (66% Se for 93% Sp)^(26)^. Blumberg et al., when comparing VF tests, OCT, and stereophotographs, demonstrated higher concordance among glaucoma specialists and higher diagnostic ability when using OCT alone for differentiating patients with suspected glaucoma and patients with early glaucoma (ĸ, 0.4)^(14)^. Lindbohm et al. showed that functional (VF) and optic nerve structural assessment (through OCT and GDx) by glaucoma specialists provided a better diagnostic accuracy compared with that obtained using VF test assessments alone^(27)^. A moderate-to-good concordance (ĸ, 0.51-0.73) among glaucoma specialists was reported for OCT rating of glaucoma or healthy patients, with an Se ranging from 76% to 79% and an Sp ranging from 68% to 81%^(28)^. One of the limitations of the present study was that an analysis made by a group of glaucoma specialists was not included for comparison. If this group were to be included, a better diagnostic performance would be expected with the addition of OCT in GIV, in addition to a better Se in GI, as previously reported^(14,28)^.

Another limitation of the present study was the randomization of 80 patients, without taking individuals’ previous glaucoma severity into consideration. A possible way to avoid this difference among groups would be to distribute patients according to their glaucoma severity based on the Hodapp-Parrish-Anderson criteria^(29)^, and later performing a separate randomization of these subsets, with the same number of patients with each level of severity being distributed among the four groups.

Another reason for the poor GIV performance may have been the order in which the slides were shown, i.e., always in the same sequence, GI-GII-GIII-GIV. This could have generated fatigue and reduced the attention of examiners toward the end of the analysis of 80 slides, potentially impairing their performance in terms of correct diagnoses of the last group. However, there was no time limit for the analysis, and it was not mandatory that assessments should occur in the same order as the slides were shown.

Another limitation was that the complementary test diagnostic performance was assessed in a sample known to comprise 50% patients with glaucoma, which may have overestimated the diagnostic ability among examiners.

Although OD assessment through stereophotographs is considered to be the “gold standard”^(30)^, CR was chosen for OD and RNFL analysis in this study. The latter approach was deemed the most appropriate for the proposed methodology: remote image analysis of slides that were emailed to evaluators. In addition, it offered greater convenience in terms of image acquisition and is a test with a larger reach among non-glaucoma specialists, thereby facilitating the analysis. A previous study found that inter-examiner concordance in glaucoma diagnosis using CR (ĸ, 0.61) was not inferior to that obtained using stereophotographs (ĸ, 0.59)^(31)^. Thus, the choice of using CR was not regarded as a limitation of this study.

It may not be possible to extrapolate the results found here to a population with characteristics different from that studied (50% of which were glaucoma patients). Moreover, the participating ophthalmologists used only complementary tests to make a diagnosis, without direct patient examination, assessment of the contralateral eye, or access to a patient’s chart and associated clinical data.

Finally, we concluded that CR analysis alone fails to effectively allow or exclude accurate glaucoma diagnosis. However, combining VF or OCT assessments with CR improved its usefulness for diagnosing glaucoma.
